# Farnesoid X Receptor Ligand Prevents Cisplatin-Induced Kidney Injury by Enhancing Small Heterodimer Partner

**DOI:** 10.1371/journal.pone.0086553

**Published:** 2014-01-27

**Authors:** Eun Hui Bae, Hong Sang Choi, Soo Yeon Joo, In Jin Kim, Chang Seong Kim, Joon Seok Choi, Seong Kwon Ma, JongUn Lee, Soo Wan Kim

**Affiliations:** 1 Department of Internal Medicine, Chonnam National University Medical School, Gwangju, Korea; 2 Department of Physiology, Chonnam National University Medical School, Gwangju, Korea; Shanghai Jiao Tong University School of Medicine, China

## Abstract

The farnesoid X receptor (FXR) is mainly expressed in liver, intestine and kidney. We investigated whether 6-ethyl chenodeoxycholic acid (6ECDCA), a semisynthetic derivative of chenodeoxycholic aicd (CDCA, an FXR ligand), protects against kidney injury and modulates small heterodimer partner (SHP) in cisplatin-induced kidney injury. Cisplatin inhibited SHP protein expression in the kidney of cisplatin-treated mice and human proximal tubular (HK2) cells; this effect was counteracted by FXR ligand. Hematoxylin and eosin staining revealed the presence of tubular casts, obstructions and dilatations in cisplatin-induced kidney injury, which was attenuated by FXR ligand. FXR ligand also attenuated protein expression of transforming growth factor-β1 (TGF-β1), Smad signaling, and the epithelial-to-mesenchymal transition process, inflammatory markers and cytokines, and apoptotic markers in cisplatin-treated mice. Cisplatin induced NF-κB activation in HK2 cell; this effect was attenuated by pretreatment with FXR ligand. In SHP knockdown by small interfering RNA, cisplatin-induced activation of TGF-β1, p-JNK and Bax/Bcl-2 ratio was not attenuated, while SHP overexpression and FXR ligand inhibited expression of these proteins in cisplatin-pretreated HK2 cells. In conclusion, FXR ligand, 6ECDCA prevents cisplatin-induced kidney injury, the underlying mechanism of which may be associated with anti-fibrotic, anti-inflammatory, and anti-apoptotic effects through SHP induction.

## Introduction

Cisplatin is one of the most frequently used chemotherapeutic agents against solid tumors [1]. However, clinical use may be limited by its potential nephrotoxicity. We have demonstrated that cisplatin-induced nephrotoxicity is associated with the activation of apoptosis, inflammation and fibrosis pathways [2]. A single injection of cisplatin may induce renal epithelial cell damage, epithelial-to-mesenchymal transition (EMT), and progressive interstitial fibrosis, along with an upregulation of transforming growth factor-β1 (TGF-β1) [3], [4]. Cisplatin may activate pro-apoptotic genes and repress anti-apoptotic genes by transcriptional regulation [5].

Farnesoid X receptor (FXR) is a member of the nuclear receptor superfamily of ligand-activated transcription factors that functions as an endogenous sensor for bile acids [6]. The most potent natural activator of human FXR seems to be chenodeoxycholic acid (CDCA). 6-Ethyl chenodeoxycholic acid (6ECDCA), which increases FXR affinity by substitution with alkyl groups is a more potent FXR agonist than CDCA [7]. It has been recently reported that FXR ligand has renoprotective effects in high-fructose-fed wistar rats [8], and diabetic nephropathy [9]. However, the molecular basis of this phenomenon has not been defined.

The orphan nuclear receptor small heterodimer partner (SHP) is a well-known FXR target and metabolic regulator [10], [11]. It is an atypical member of the orphan nuclear receptor superfamily because it lacks a DNA-binding domain but contains a putative ligand binding domain [12]. Recent studies have shown that loss of SHP accelerates renal fibrosis after unilateral ureter obstruction [13], and demonstrated that SHP is an intrinsic negative regulator of Toll-like receptor-triggered inflammation in endotoxin-induced sepsis [14].

We investigated whether FXR ligand could attenuate fibrosis, inflammation and apoptosis in cisplatin-induced kidney injury. Furthermore, we assessed whether the therapeutic effects of FXR ligand were modulated by SHP knockdown or overexpression.

## Materials and Methods

### Animals

The experimental protocol was approved by the Institutional Animal Care and Use Committee of Chonnam National University Medical School. Male 8-wk-old C57BL6 mice purchased from Samtako (Korea). The mice were treated with cisplatin alone (20 mg/kg, single injection, *i.p.*; Boryung, Ansan, Korea; n = 9) or cisplatin+FXR ligand (30 mg/kg/day, for 7 days, *per oral*; Santa Cruz; Califonia, USA; n = 9). Cisplatin was dissolved in normal saline. The control group received the vehicle only (physiologic saline, n = 9). On the experimental day, the mice were anesthetized with 2% isoflurane and 100% oxygen. Blood samples were then collected from the left ventricle. The right kidney was rapidly removed, and then processed for semiquantitative immunoblotting. The left kidney was fixed via retrograde perfusion for immunohistochemistry.

Another series of experiment was done for the assay of real-time polymerase chain reaction (PCR). The mice were decapitated under a conscious state, and their kidneys were taken and kept at −70°C until assayed for the mRNA expression by real time-PCR.

### Histological Examination

A perfusion needle was inserted into the abdominal aorta, and the vena cava was cut to establish an outlet. Blood was flushed from the kidney with cold phosphate-buffered saline (PBS, pH 7.4) for 15 s before switching to ice-cold 3% paraformaldehyde in PBS (pH 7.4) for 3 min. The kidney was removed and cut into 2- to 3-mm-thick transverse sections, which were immersion fixed for 1 h, followed by three 10-min washes in PBS. The tissue was dehydrated in a graded ethanol series and incubated in xylene overnight. After embedding the tissue in paraffin, 2-µm-thick sections were cut with a rotary microtome (Leica Microsystems; Herlev, Denmark), and stained with hematoxylin and eosin (H&E) for histological examination. Immunoperoxidase labeling was conducted as described previously [15].

### Real-Time Polymerase Chain Reaction (Real-Time PCR)

Renal cortex was homogenized in Trizol reagent (Invitrogen, Carlsbad, CA). RNA was extracted with chloroform, precipitated with isopropanol, washed with 75% ethanol, and then dissolved in distilled water. The RNA concentration was determined by the absorbance read at 260 nm (Ultraspec 2000; Pharmacia Biotech, Cambridge, UK). The mRNA expression of inflammatory cytokines and adhesion molecules was determined by real-time PCR. cDNA was made by reverse transcribing 5 µg of total RNA using oligo(dT) priming and superscript reverse transcriptase II (Invitrogen, Carlsbad, CA). cDNA was quantified using Smart Cycler II System (Cepheid, Sunnyvale, CA) and SYBR Green was used for detection. Each PCR reaction was done in 10 µM forward primer, 10 µM reverse primer, 2X SYBR Green Premix Ex Taq (TAKARA BIO INC, Seta 3-4-1, Japan), 0.5 µl cDNA and H_2_O to bring the final volume to 20 µl. Relative levels of mRNA were determined by real-time PCR, using a Rotor-GeneTM 3000 Detector System (Corbette research, Mortlake, New South Wales, Australia). Sequences of primers are listed in Table 1.

**Table 1:** Primer sequences for real-time PCR.

Gene	Sequence	Size, bp
TNF-α	Fwd: GCATGATCCGCGACGTGGAA	352
	Rev: AGATCCATGCCGTTGGCCAG	
IL-1β	Fwd: TGA TGT TCC CAT TAG ACA GC	378
	Rev: GAG GTG CTG ATG TAC CAG TT	
MCP-1	Fwd: CACCTGCTGCTACTCATTCACT	349
	Rev: AGAAGTGACCAGTATGACAGAGAAC	
ICAM-1	Fwd: GCCCGGAGGATCACAAACGAC	186
	Rev: CCTGGGGCTGGCATGTAAGAGT	
GAPDH	Fwd: GCCAAAAGGGTCATCATCTC	229
	Rev: GGCCATCCACAGTCTTCT	

TNF, tumor nectoric factor; interleukin, IL; MCP, monocyte chemoattractant protein; ICAM, intercellular adhesion molecule; Fwd, forward; Rev, reverse.

The PCR was performed according to the following steps: 1) 95°C for 5 min; 2) 95°C for 20 s; 3) 58 to 62°C for 20 s (optimized for each primer pair); 4) 72°C for 30 s; and 5) 85°C for 6 s to detect SYBR Green. Steps 2–5 were repeated for additional 64 cycles, while at the end of the last cycle temperature was increased from 60 to 95°C to produce a melt curve. Data from the reaction were collected and analyzed with the Corbett Research Software. The comparative critical threshold (Ct) values from quadruplicate measurements were used to calculate the gene expression, with normalization to GAPDH as an internal control [16]. Melting curve analysis was performed to enhance specificity of the amplification reaction.

### Semiquantitative Immunoblotting

Kidney tissues were homogenized in ice-cold isolation solution containing 0.3 M sucrose, 25 mM imidazole, 1 mM ethylenediamine tetraacetic acid (EDTA), 8.5 µM leupeptin, and 1 mM phenylmethylsulfonyl fluoride (pH 7.2). The homogenates were centrifuged at 1000×*g* for 15 min at 4°C to remove whole cells, nuclei, and mitochondria. The total protein concentration was measured by bicinchoninic acid (BCA) assay kit (Pierce; Rockford, IL, USA). All samples were adjusted to reach the same final protein concentrations. They were then dissolved at 65°C for 15 min in SDS-containing sample buffer and stored at −20°C. To confirm equal loading of proteins, an initial gel was stained with Coomassie blue. SDS-PAGE was performed on 9 or 12% polyacrylamide gels. The proteins were electrophoretically transferred onto nitrocellulose membranes (Hybond ECL RPN3032D; Amersham Pharmacia Biotech; Little Chalfont, UK) using Bio-Rad Mini Protean II apparatus (Bio-Rad; Hercules, CA, USA). The blots were blocked with 5% milk in PBS-T (80 mM Na_2_HPO_4_, 20 mM NaH_2_PO_4_, 100 mM NaCl, and 0.1% Tween-20 at pH 7.5) for 1 h; incubated overnight at 4°C with primary antibodies; and incubated with secondary anti-rabbit, anti-mouse, or anti-goat horseradish peroxidase-conjugated antibodies thereafter. The immunoblots were then visualized using an enhanced chemiluminescence system.

### Terminal Deoxynucleotidyl Transferase-Mediated dUTP Nick-End Labeling (TUNEL) Assay

The ApopTag in situ apoptosis detection kit (Oncor, Gaithersburg, MD) was used. The sections were dewaxed and treated with proteinase K, then incubated with equilibration buffer for 10 min, followed by incubation with working-strength TdT enzyme solution at 37°C for 2 hr. The reaction was terminated by incubation in working-strength stop/wash buffer for 30 min at 37°C. Sections were then incubated with antidigoxigenin peroxidase and then incubated with diaminobenzidine and 0.01% H_2_O_2_ for 5 min at room temperature. The sections were counterstained with hematoxylin and examined by light microscopy [17].

### Nuclear Extracts Preparation

For nuclear extracts, cells were lysed using NE-PER® nuclear extraction reagent (NER) (Pierce Biotechnology, Rockford, IL, USA) according to the manufacturer’s protocol. Briefly, HK-2 cells incubated with cisplatin were harvested by scraping into cold PBS, pH 7.2 and then centrifuged at 14,000×*g* for 2 min. After removing the supernatant, 100 µL of ice-cold cytoplasmic extraction reagent (CER) I was added to the dried cell pellets. After incubated on ice for 10 min, ice-cold CER II was added to the tube. The tube was centrifuged at 16,000×*g* for 5 min and pellet fraction was suspended in 50 µL of ice-cold NER. After centrifuging the tube at 16,000×*g* for 10 min, the supernatant (nuclear extract) fraction was transferred to a clean tube [18], [19].

### Cell Culture

HK-2 cells (ATCC; Manassas, VA, USA) were passaged every 3–4 days in 100-mm dishes containing combined Dulbecco’s modified Eagle’s (DMEM) and Hams F-12 medium (Sigma) supplemented with 10% fetal bovine serum (FBS; Life Technologies; Gaithersburg, MD, USA), 100 U/ml penicillin, and 100 mg/ml streptomycin (Sigma). The cells were then incubated in a humidified atmosphere of 5% CO_2_ and 95% air at 37°C for 24 h, and sub-cultured until 70–80% confluence. Cells were plated onto 60-mm dishes in a medium containing 10% FBS and incubated for 24 h, following which they were transferred to DMEM-F12 medium with 2% FBS and incubated for an additional 16 h. The cells were then treated with cisplatin (50 µM), either with or without 6-ECDCA (20 µM). The control cells were treated with a buffer solution alone.

### siRNA Experiments

For knockdown of SHP expression, siRNA for SHP were chemically synthesized (Dharmacon.INC, Chicago, USA) and transfected according to the manufacturer’s instructions. HK2 cells were transfected with siRNA using DhamaFECT 2 reagent (Dharmacon.INC, Chicago, USA). Efficiency of knockdown was performed through Western blot analysis.

### Plasmid Construct and Transfection

pcDNA3-mSHP was kindly provided by Prof. Heung-Sik Choi (Chonnam National University, South Korea). The mouse SHP was subcloned into NcoI/*Xho*I site of pcDNA3 vector [20]. pcDNA3-mSHP or pcDNA3 was introduced to HK2 cells by FuGene HD reagent (promega, USA). Two days after transfection, we identified the overexpression of SHP and Flag in HK2 cells by Western blot analysis.

### Primary Antibodies

Anti-rabbit antibodies against TGF-β1 (polyclonal; Santa Cruz Biotechnology, Santa Cruz, CA, USA), extracellular signal-regulated kinases 1/2 (ERK 1/2), anti-phosphorylated ERK (p-ERK 1/2), anti-c-Jun N-terminal kinase (JNK), anti-phosphorylated JNK (p-JNK; monoclonal; Cell Signaling Technology, MA, USA), E-cadherin (BD Transduction Laboratories, San Jose, CA, USA), connective tissue growth factor (CTGF, Santa Cruz), Bax, Bcl2, Smad-2/3, Smad-4, Smad-6, cleaved caspased-3 (Cell Signaling Technology, MA, USA), and phospho-NF-kB p65 (p65 NF-κB; Ser536; monoclonal; Cell Signaling Technology, MA, USA), anti-mouse antibodies against ED-1 (monoclonal; abcam lnc, MA, USA) and α-SMA (1A4 Clone; monoclonal; Sigma Chemical Co. St. Louis, MO, USA) and fibronectin (2Q604; monoclonal; Santa Cruz Biotechnology, Santa Cruz, CA, USA) were commercially obtained.

### Statistical Analysis

The results were expressed as mean ± SEM. Multiple comparisons among the 3 groups were performed using one-way ANOVA and the *post-hoc* Tukey’s honestly significant difference test. Differences with values of *p*<0.05 were considered significant.

## Results

### FXR Ligand Attenuated Cisplatin-Induced Downregulation of SHP

**Figure 1:**
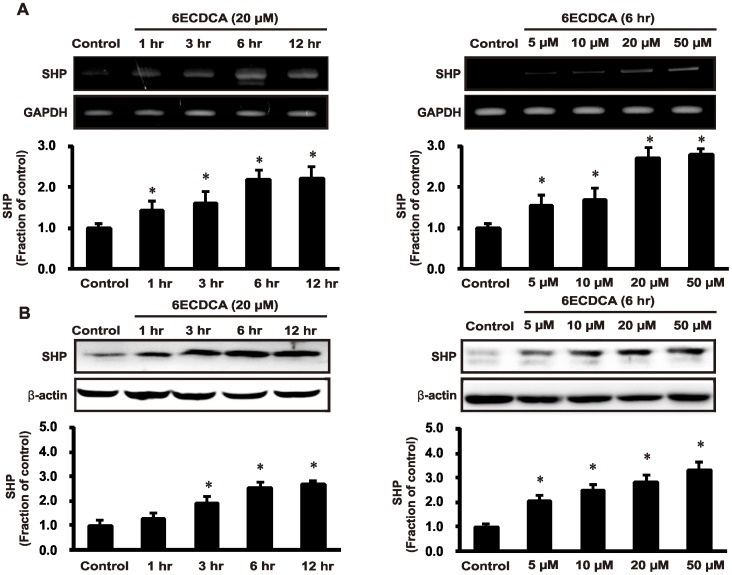
Time- and dose-dependent SHP mRNA and protein expression after FXR ligand 6ECDCA treatment in HK-2 cells. (A) mRNA expression of SHP was increased with 6ECDCA treatment. (B) SHP protein expression was increased by treatment with 6ECDCA. Each column represents mean ± SEM. 6ECDCA, 6-Ethyl chenodeoxycholic acid; SHP, small heterodimer partner. GAPDH and β-actin protein levels were analyzed as internal controls. *p<0.05, compared with the control. Data are representative of at least 3 independent experiments.

**Figure 2:**
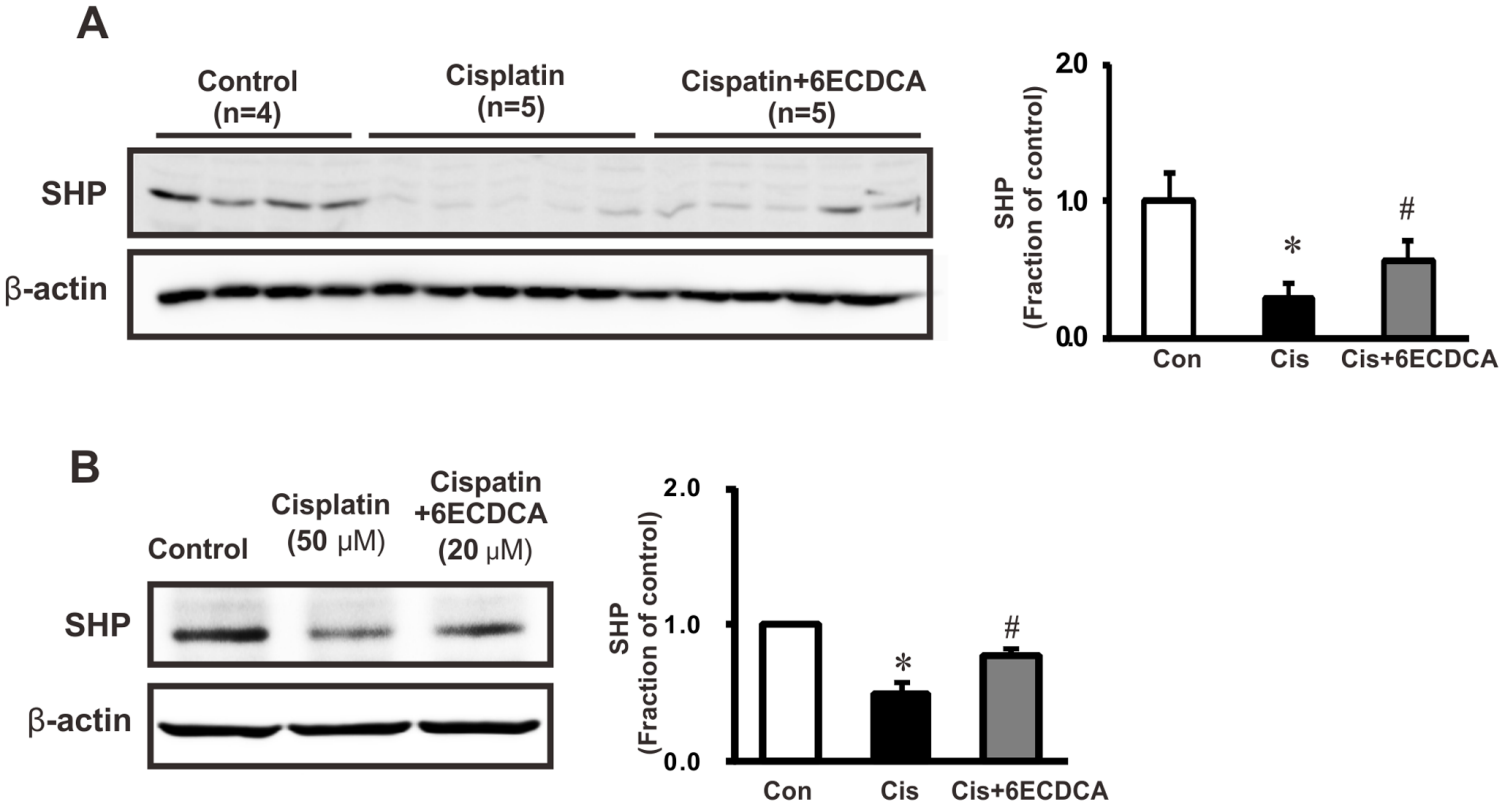
Immunoblotting of SHP in cisplatin-induced renal injury. SHP protein expression was reduced in cisplatin-induced renal injury mice, and it was attenuated by FXR ligand treatment (A). In HK-2 cells, SHP protein exhibited significantly decreased expression after cisplatin (50 µM, 16 h) treatment, and FXR ligand (20 µM, 1 h) pretreatment prevented this cisplatin-mediated decrease in SHP expression (B). Each column represents mean ± SEM. 6ECDCA, 6-ethyl chenodeoxycholic acid; SHP, small heterodimer partner. β-actin protein served as an internal control. Each column represents mean ± SEM. *p<0.05, compared with the control. #*p*<0.05, compared with the cisplatin treatment.

To investigate whether FXR ligand 6ECDCA induces SHP expression, we treated human proximal tubular epithelial (HK2) cells with 6ECDCA. FXR ligand yielded time- and dose-dependent increases in mRNA and protein expression of SHP in HK2 cells (Figure 1). Moreover, SHP expression was inhibited in cisplatin-induced kidney injury in mice; this effect was attenuated by FXR ligand (Figure 2A). Moreover, we analyzed the protein expression of SHP by immunoblotting in HK-2 cells following pretreated with FXR ligand (20 µM) for 1 h and subsequent treatment with 50 µM cisplatin for 16 h. SHP protein exhibited significantly decreased expression after cisplatin treatment, and FXR ligand pretreatment prevented this cisplatin-mediated decrease in SHP expression (Figure 2B).

### FXR Ligand Attenuated Functional and Morphological Changes in Cisplatin-Induced Kidney Injury

**Figure 3:**
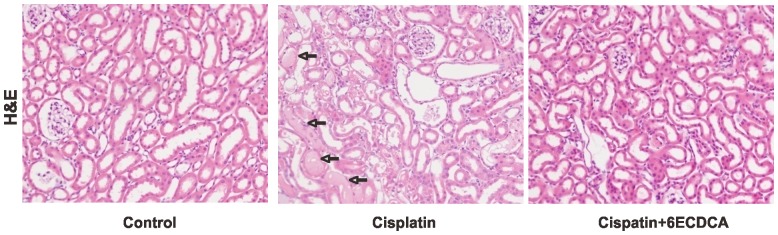
Effects of FXR ligand treatment on morphological changes in cisplatin-induced kidney injury. Hematoxylin and eosin (H&E) staining revealed the presence of tubular casts and obstructions (arrow) in the kidney of cisplatin-treated mice, which was attenuated by FXR ligand co-treatment. Magnification,×200.

Table 2 represents changes in functional parameters. Cisplatin treatment increased plasma levels of BUN and creatinine. FXR ligand co-treatment significantly ameliorated these cisplatin-induced renal changes. Hematoxylin and eosin staining revealed the presence of tubular casts and obstructions in cisplatin-induced kidney injury, which was attenuated by FXR ligand (Figure 3).

**Table 2:** Renal functional parameters.

	Control(n = 9)	Cisplatin(n = 9)	Cisplatin+6-ECDCA (n = 9)
Body weight (g)	26.2±0.06	21.6±0.03*	22.5±1.26*
P_Cr_ (mg/dl)	0.27±0.05	2.08±0.1.40*	0.38±0.15#
BUN (mg/dl)	26.97±1.94	174.58±31.89*	69.48±45.06* ^,^#

Abbreviations: *n*, number of rats; P_Cr_, plasma creatinine; BUN, blood urea nitrogen.

* *p*<0.05, compared with control.

#*p*<0.05, compared with the cisplatin-treated mice. Values are means ± SEM.

### FXR Ligand Inhibits TGF-β1 and Smad Stimulated Extracellular Matrix Protein Expression

**Figure 4:**
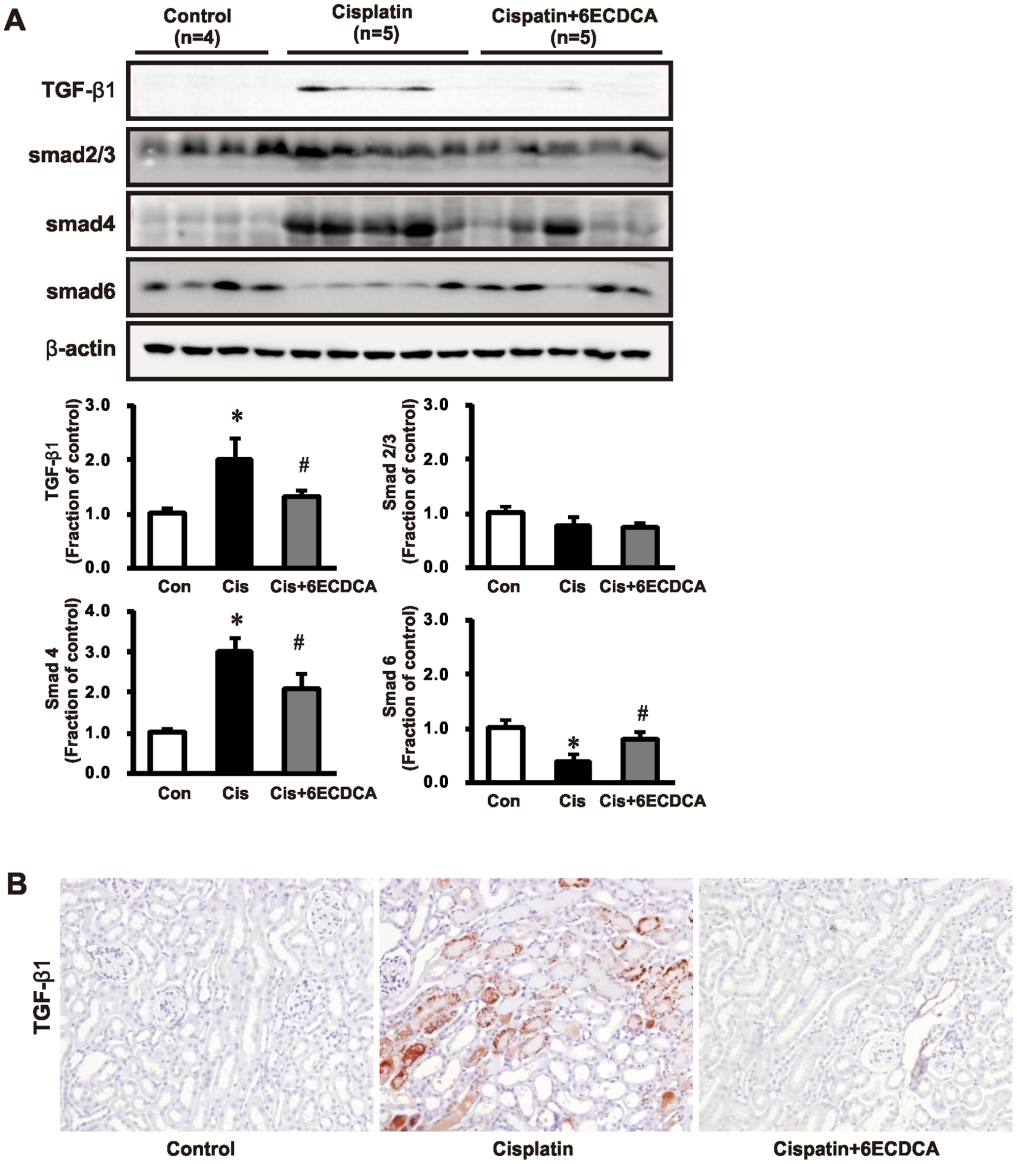
Effects of FXR ligand on TGF β1-Smad signaling in cisplatin-induced kidney injury. (A) TGF-β1 expression was increased in the cisplatin group. Cisplatin also increased the expression of Smad-4, while it reduced the level of inhibitory Smad-6. These cisplatin-induced changes were abolished or significantly attenuated in the FXR ligand-co-treated group. Each column represents mean ± SEM. *p<0.05, compared with the control. #*p*<0.05, compared with the cisplatin treatment. (B) Immunoperoxidase microscopy of TGF-β1 in the kidney cortex. Increased immunolabeling was evident in the cisplatin-treated mouse kidneys; the increase was prevented by FXR ligand treatment. Magnification,×200.

**Figure 5:**
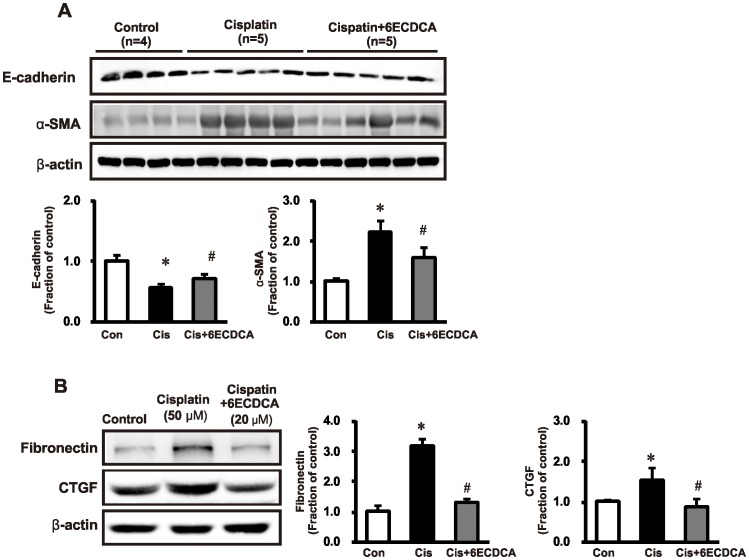
Effects of FXR ligand on epithelial-mesenchymal transition and fibrosis in cisplatin-induced kidney injury. (A) Protein expression of the epithelial receptor E-cadherin and the myofibroblast molecular marker α-SMA was also assessed. In cisplatin-treated mice, E-cadherin expression decreased and α-SMA increased; this effect was prevented in the FXR ligand-co-treated group. (B) *In vitro* (HK-2 cells) studies showed that FXR ligand pretreatment (20 µM, 1h) also reduced fibronectin and connective tissue growth factor (CTGF) expression induced by cisplatin (50 µM, 16 h). Each column represents mean ± SEM. *p<0.05, compared with the control. #*p*<0.05, compared with the cisplatin treatment.

We investigated the effects of FXR ligand on TGF-β1-Smad signaling in cisplatin-induced injury. Figure 4 shows the expression of TGF-β1 and Smad proteins. TGF-β1 expression increased in the cisplatin group. Cisplatin also increased the expression of total Smad-4, and reduced expression of inhibitory Smad-6. These cisplatin-induced changes were abolished or significantly attenuated by FXR ligand co-treatment. Expression of the epithelial receptor E-cadherin and the myofibroblast molecular marker α-SMA was also assessed. In cisplatin-treated mice, E-cadherin expression decreased and α-SMA increased, which was also prevented by co-treatment with FXR ligand (Figure 5A). We performed *in vitro* to confirm *in* *vivo* data. *In vitro* studies showed that FXR ligand pretreatment (20 µM for 1 h) also counteracted cisplatin (50 µM for 16 h) -induced upregulation of fibronectin and connective tissue growth factor (CTGF) (Figure 5B).

### FXR Ligand Attenuates Inflammatory Cytokines and Adhesion Molecules in Cisplatin-Induced Kidney Injury

**Figure 6:**
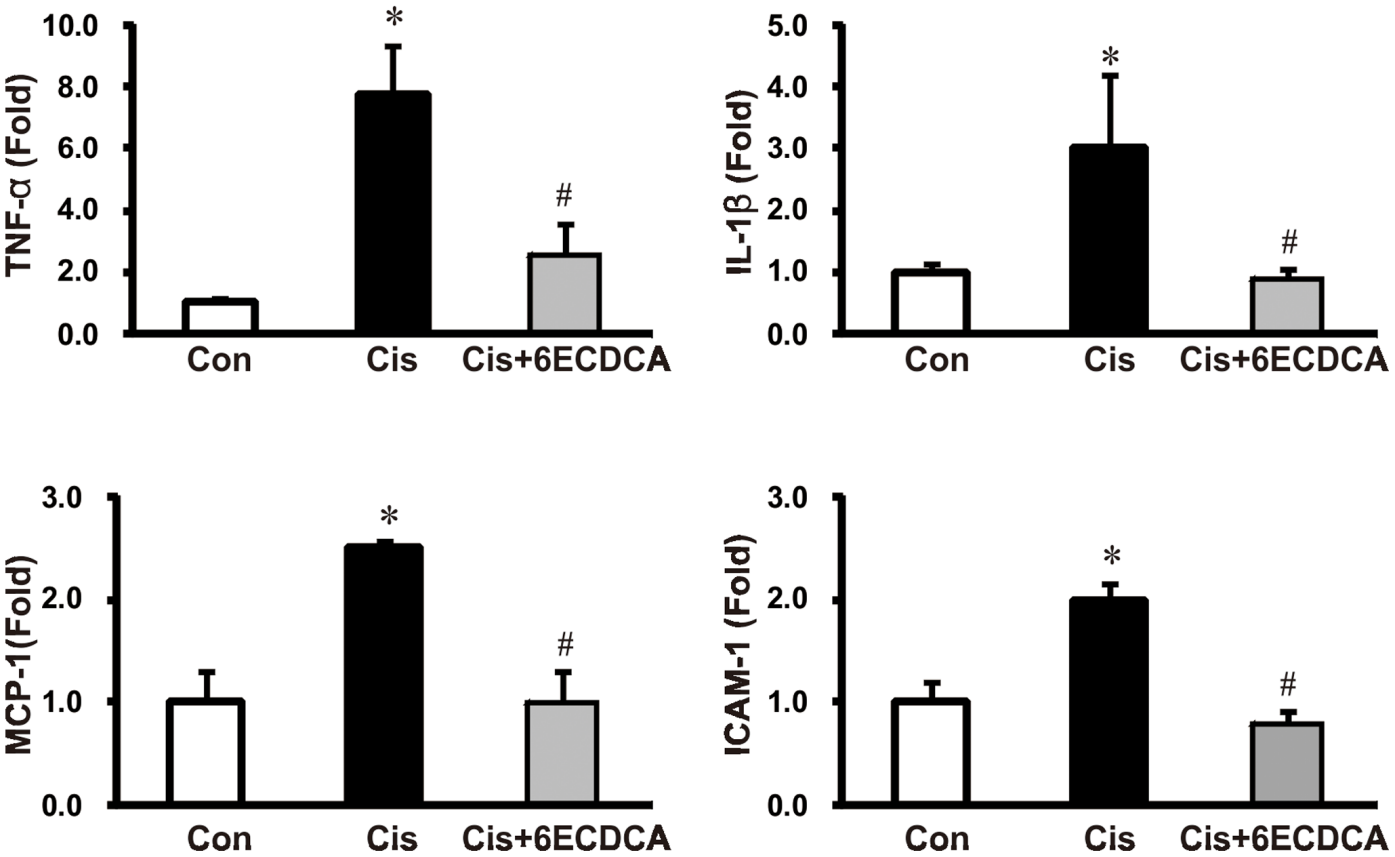
Effects of FXR ligand on inflammatory cytokines and adhesion molecules in cisplatin-induced kidney injury. Cisplatin treatment significantly induced renal TNF-α, IL-1β, MCP-1, and ICAM-1 mRNA expression. FXR ligand co-treatment suppressed the overexpression of these inflammatory cytokines and adhesion molecules. Each column represents mean ± SEM. *p<0.05 compared with the control animals. #*p*<0.05 compared with the cisplatin-treated mice.

We also investigated the expression of TNF-α and IL-1β, which are key inflammatory cytokines produced by infiltrating cells. As shown in Figure 6, cisplatin significantly induced renal TNF-α and IL-1β mRNA expression, while these changes were attenuated with FXR-ligand co-treatment. We also observed increased expression of certain chemokines and adhesion molecules such as MCP-1 and ICAM-1, which can activate, recruit, or induce the transmigration of inflammatory cells to the site of kidney injury. Expression of these factors was induced by cisplatin treatment. FXR ligand co-treatment significantly reduced expression of these chemokines in cisplatin-treated mouse kidneys (Figure 6).

### FXR Ligand Attenuates Inflammatory Proteins and the MAPK pathway in Cisplatin-Induced Kidney Injury

**Figure 7:**
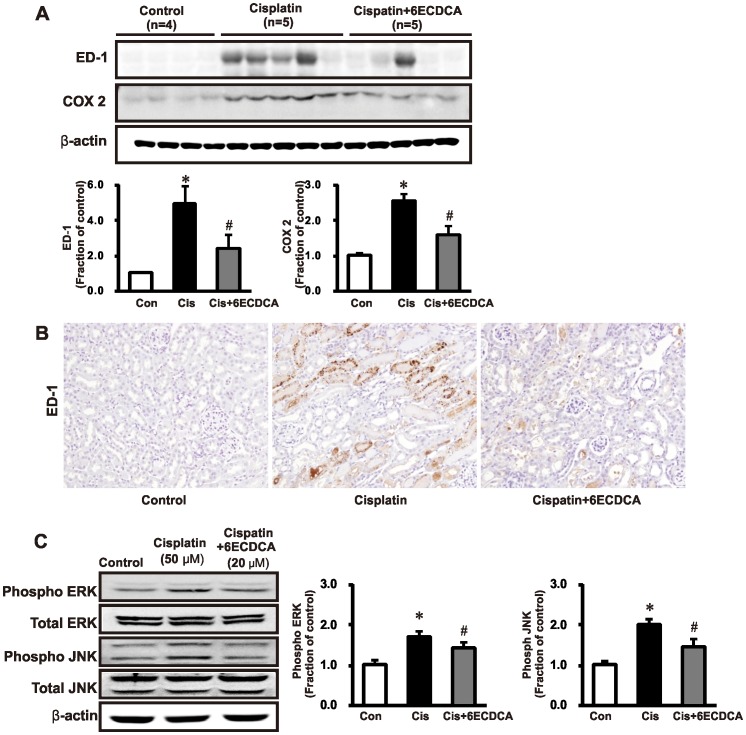
Effects of FXR ligand on inflammatory proteins and MAPK pathway in cisplatin-induced kidney injury. (A) ED-1 and COX2 protein expression in the kidney was significantly higher in cisplatin-treated mice than in the controls, which was ameliorated by FXR ligand treatment. Each column represents mean ± SEM. *p<0.05 compared with the control animals. #*p*<0.05 compared with cisplatin-treated mice. (B) The infiltration of ED-1-positive macrophages in cisplatin-treated mice was significantly higher than in control mice. FXR ligand co-treatment abrogated inflammatory cell infiltration in cisplatin-treated kidneys. Magnification, 200×. (C) HK-2 cells were incubated with cisplatin (50 µM. 3 h) and pERK and pJNK expression were determined in cells pretreated with FXR ligand (20 µM, 1 h) before cisplatin administration. In this experiment, pERK and pJNK overexpression induced by cisplatin was significantly repressed by FXR ligand pretreatment.

We investigated Cluster of Differentiation 68 (CD68, ED-1) and cyclooxygenase-2 (COX2) as inflammatory proteins markers. Protein expression of ED-1 and COX2 in the kidney was higher in cisplatin-treated mice than in controls, and these effects were reversed by FXR ligand (Figure 7A). Infiltration of ED-1-positive macrophages was higher in cisplatin-treated mice than in the control mice. FXR ligand co-treatment abrogated inflammatory cell infiltration in cisplatin-treated kidneys (Figure 7B). As shown in Figure 7C, phosphorylation of ERK1/2, and JNK increased following treatment with cisplatin (50 µM for 2 h) in HK-2 cells, which was attenuated by pretreated with FXR ligand (20 µM) at 1 h before cisplatin administration. In this experiment, cisplatin-induced pERK and pJNK overexpression was significantly repressed by pretreatment with FXR ligand.

### FXR Ligand Attenuates Cisplatin-Induced Renal Tubular Cell Apoptosis

**Figure 8:**
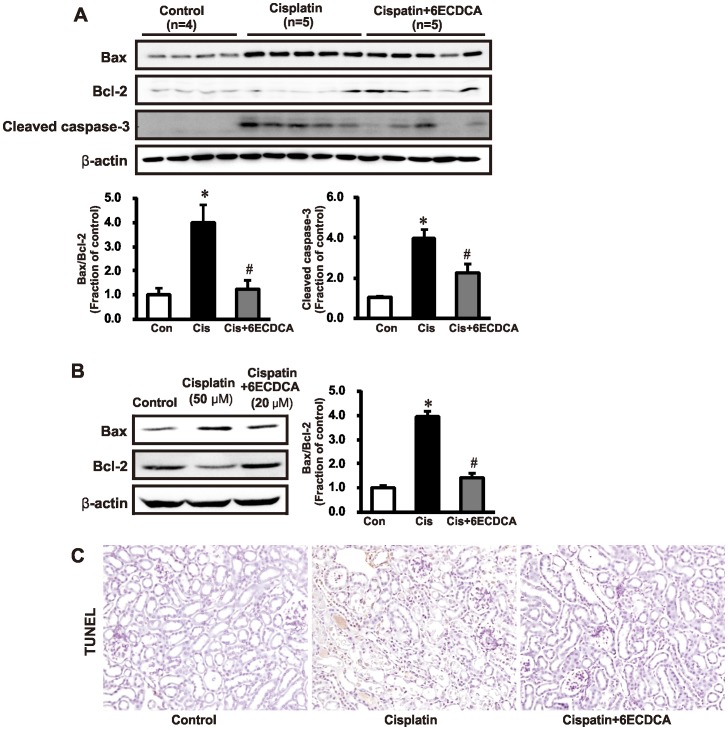
Effects of FXR ligand on apoptosis in cisplatin-induced kidney injury. (A) Cisplatin treatment increased expression of the pro-apoptotic marker Bax, cleaved caspase-3 and decreased expression of the antiapoptotic protein Bcl-2, resulting in an overall increase in the Bax/Bcl-2 ratio. FXR ligand treatment attenuated the increase in Bax/Bcl-2 ratio and cleaved caspase-3 in cisplatin-treated mice. *p<0.05 compared with the control animals. #*p*<0.05 compared with cisplatin-treated mice. (B) Bax/Bcl-2 increased in cisplatin (50 µM, 24 h)-treated HK-2 cells, and the effect was attenuated by FXR ligand (20 µM, 1 h) pretreatment. *p<0.05 compared with the control. #*p*<0.05 compared with cisplatin-treated HK-2 cells. (C) The terminal deoxynucleotidyl transferase-mediated dUTP nick end-labeling (TUNEL) assay showed increased apoptosis in response to cisplatin, whereas FXR ligand co-treatment significantly reduced the number of TUNEL-positive cells. Magnification,×200.

Cisplatin increased expression of the proapoptotic marker Bax and inhibited the anti-apoptotic protein Bcl-2, resulting in an overall increase in the Bax/Bcl-2 ratio. FXR ligand attenuated the increase in the Bax/Bcl-2 ratio in cisplatin-treated mice (Figure 8A). Furthermore, Bax/Bcl-2 increased in cisplatin (50 µM, 24 h)-treated HK-2 cells, and was attenuated by FXR ligand (20 µM, 1hr) pretreatment (Figure 8B). To determine the protective effects of FXR ligand in cisplatin-induced renal tubular apoptosis, we performed terminal deoxynucleotidyl transferase-mediated dUTP nick-end labeling (TUNEL). The number of tubular epithelial cells containing TUNEL-positive nuclei increased in cisplatin-treated mouse kidneys, while FXR ligand co-treatment attenuated this effect (Figure 8C).

### FXR Ligand Attenuates NF-κB Expression in Cisplatin-Induced HK2 Cell Injury

**Figure 9:**
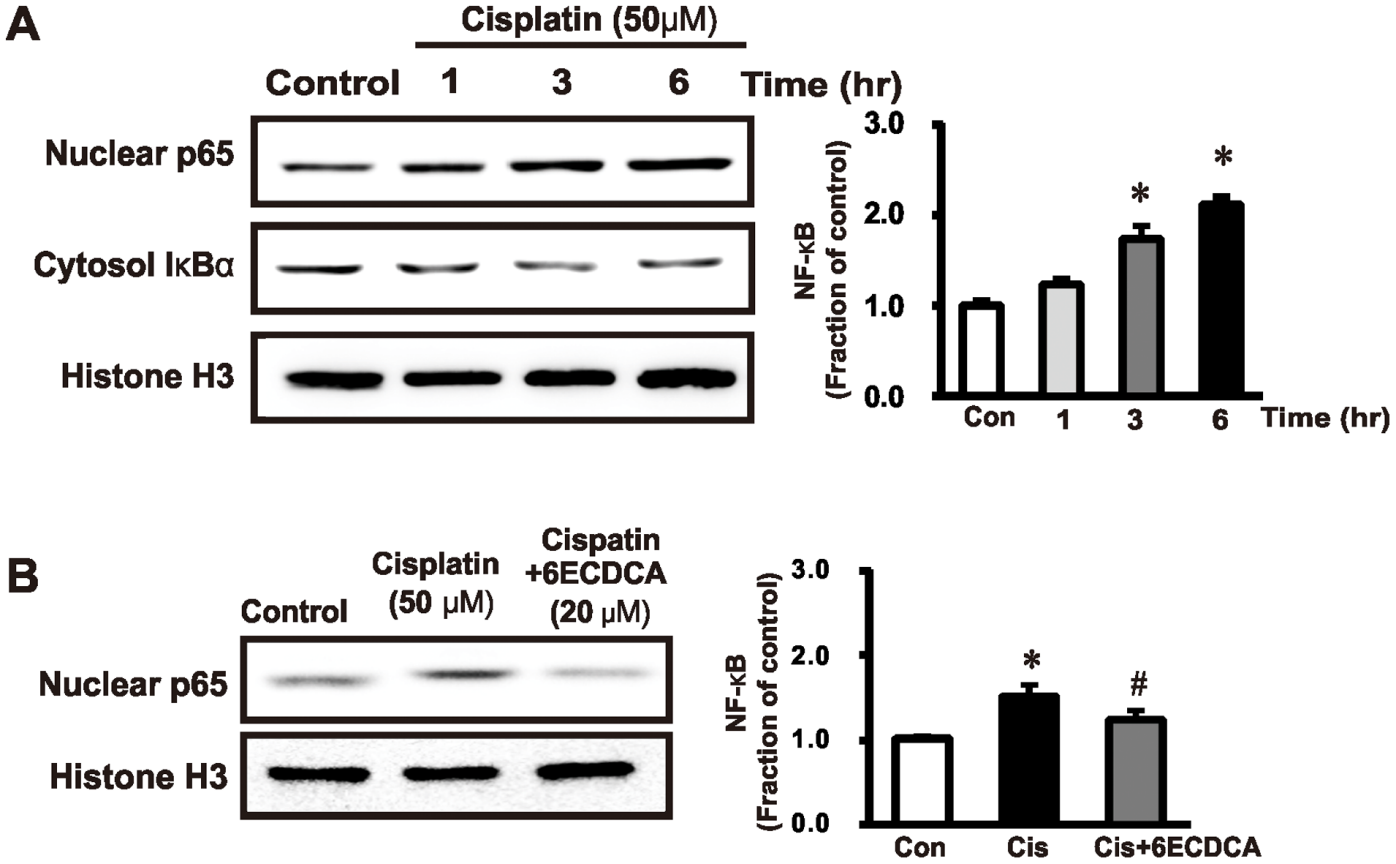
Effects of FXR ligand on NF-κB expression in HK 2 cells. (A) Expression of the p65 subunit of nuclear factor κB (NF-κB) and cytosol IκBα was assessed by semiquantitative immunoblotting in HK-2 cells incubated with cisplatin (50 µM). Nuclear p65 subunit expression was increased by cisplatin (50 µM, 3h). (B) FXR ligand (20 µM) was used to pretreat HK-2 cells 1 h before cisplatin exposure. The cisplatin-induced overexpression of the nuclear p65 subunit of NF-κB was ameliorated with FXR ligand (20 µM, 1h) pretreatment. *p<0.05 compared with the control. #*p*<0.05 compared with cisplatin-treated HK-2 cells.

Figure 9A shows the changes in the expression of NF-κB p65 in nuclear extracts of renal tubular HK-2 cells incubated with cisplatin (50 µM). Expression of the p65 NF-κB subunit increased 3 h after cisplatin exposure. NF-κB expression was also determined in HK-2 cells pretreated with FXR ligand (20 µM) for 1 h followed by cisplatin (50 µM) exposure for 3 h. Expression of the p65 NF-κB subunit was higher with cisplatin than in the controls, while the cisplatin-induced increase was attenuated by pretreatment with FXR ligand (Figure 9B).

### Effects of FXR Ligand on Expression of TGF-β1, Apoptosis, and the MAPK Pathway in SHP-Knockdown and Overexpression-SHP HK2 Cells

**Figure 10:**
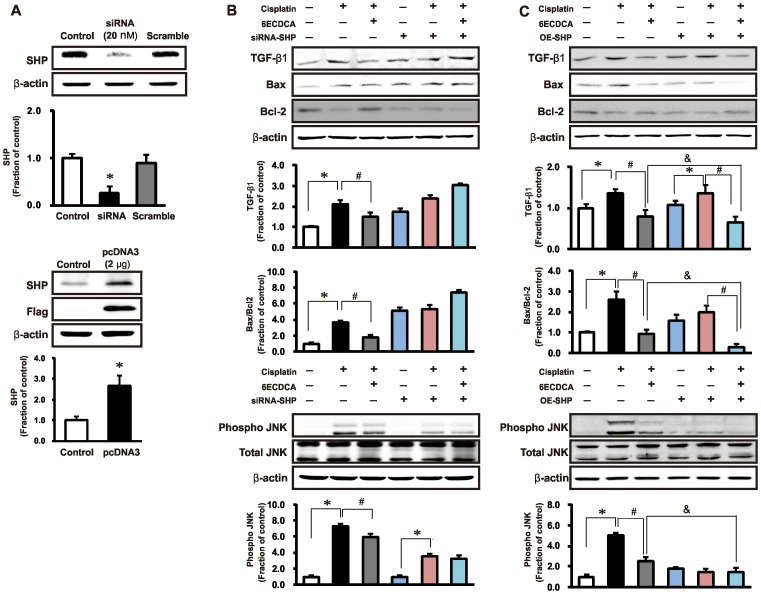
Effects of SHP knockdown or overexpression on TGF β1, Bax/bcl-2 and pJNK expression in HK 2 cells. The expression of SHP protein was decreased in SHP knockdown cells by siRNA. HK2 cells were transfected with Flag-tagged-SHP expression vector. Two days after transfection, cell lysates were immunoblotted using SHP and Flag antibodies, and their expressions were increased in SHP overexpression cells by SHP transfection (A). In HK-2 cells, cisplatin (50 µM, 3h) increased the expression of TGF-ß1, Bax/Bcl-2, and pJNK, which was partially blocked by pretreatment with FXR ligand (20 µM, 1h). In SHP knockdown cells, FXR ligand did not block cisplatin-induced overexpression of TGF-β1, Bax/Bcl-2, and pJNK (B). In SHP-overexpressing cells, FXR ligand intensified inhibition of cisplatin-induced overexpression of TGF-ß1, Bax/Bcl-2, and pJNK (C). Data are the mean ± SEM of 3 independent measurements. *p<0.05 compared with controls. #*p*<0.05 compared with cisplatin-treated HK-2 cells.^ &^ *p*<0.05 compared FXR ligand treatment with or without SHP overexpression.

As shown in Figure 10A, the expression of SHP protein was decreased in SHP knockdown cells by siRNA. HK2 cells were transfected with Flag-tagged-SHP expression vector. Two days after transfection, cell lysates were immunoblotted using SHP and Flag antibodies, and their expressions were increased in SHP overexpression cells by SHP transfection (Figure 10A). In HK-2 cells, cisplatin (50 µM) increased the expression of TGF-ß1, Bax/Bcl-2, and pJNK, which was partially blocked by pretreatment with FXR ligand (20 µM, 1h). However, in SHP knockdown cells by siRNA, FXR ligand did not block cisplatin-induced overexpression of TGF-β1, Bax/Bcl-2, and pJNK. In cells overexpessing SHP by SHP DNA transfection, FXR ligand intensified inhibition of cisplatin-induced overexpression of TGF-ß1, Bax/Bcl-2, and pJNK (Figure 10B and C).

## Discussion

The purpose of our study was to examine whether FXR ligand protects against cisplatin-induced kidney injury and whether it depends on SHP induction. Here, we demonstrated that intervention with FXR ligand, 6ECDCA significantly improves kidney injury by mediating anti-fibrosis, anti-inflammation, and anti-apoptosis processes in cisplatin-induced kidney injury. Furthermore, 6ECDCA induced SHP expression in a cisplatin nephropathy mouse model and cisplatin-treated HK2 cells. Therefore, SHP induction may be essential for FXR ligand suppression of TGF-β, P-JNK, and Bax protein expression in cisplatin-induced nephropathy. Support for this concept comes from the observation that exposure of HK 2 cells to FXR ligand increased SHP mRNA and protein, and FXR ligand is unable to suppress TGF-β, P-JNK, and Bax protein expression in SHP knocked-down HK2 cells, while their suppression was effective in SHP overexpression cells.

We found that the immediate target of FXR, SHP, was decreased in the kidneys of cisplatin- treated mice, consistent with studies in Akita and OVE26 mice, which develop diabetic nephropathy [21]. Our data are also consistent with the findings that SHP can modulate TGF-β-Smad signaling in unilateral urinary obstruction [13], and inflammation in an endotoxemia model [3].

Cisplatin, in particular, accumulates in the renal cortex, promoting interstitial proliferation of fibroblast cells and focal infiltration by inflammatory cells. In this study, cisplatin-treated rats showed a marked increase in monocyte/macrophage infiltration into the renal cortex/medulla, as indicated by the large number of ED-1-positive cells in the interstitium. Cisplatin increased the expression of proinflammatory markers such as TNF-α and IL-1β. These inflammatory molecules participate in the pathogenesis of tubulointerstitial impairment by promoting of leukocyte attraction and adhesion to inflamed renal tubular cells. The expression of cell surface adhesion molecules such as MCP-1 and ICAM-1, which are highly specific chemotactic factors for macrophages, increased in the kidneys of cisplatin-treated rats. These findings also indicate that the inflammatory process plays a significant role in the pathogenesis of cisplatin-induced renal injury. Furthermore, we showed that FXR ligand significantly reduced the infiltration of ED-1-expressing macrophages in the kidney and decreased the cisplatin-induced renal expression of proinflammatory cytokines and cell surface adhesion molecules. These findings suggest FXR ligand can attenuate kidney damage by suppression of cell surface adhesion molecules and inflammatory cytokines.

NF-κB is a key transcription factor that underlies the renal inflammatory process by regulating transcription of cytokines, chemokines, and adhesion molecules in progressive kidney diseases. In the cisplatin rat model, NF-κB was activated by cisplatin administration, and attenuation of renal NF-κB activity reduced cisplatin-induced kidney injury [22]. NF-κB is released from inhibitory subunit I-κB and translocates into the nucleus, where it promotes transcriptional activation of target genes [23]. In our study, expression of the nuclear p65 subunits of NF-κB increased after cisplatin treatment in HK-2 cells, which suggests that cisplatin induced nuclear NF-κB translocation and activation. Multiple possible intracellular mechanisms regulate inflammation and fibrosis cascades in diseased kidneys. TGF-ß1 is a key molecule in these processes, and a comprehensive survey indicates that in tubular epithelial cells, it is capable of activating several signal transduction pathways such as those involving MAPKs [24]. MAPKs are fundamental regulators of most immune cell functions, including proliferation, differentiation, survival and apoptosis, chemoattraction, and inflammatory mediator production. They use parallel signal transduction pathways, including ERK, c-Jun NH2-terminal kinase (JNK), and p38. The JNK signaling pathway is negatively regulated by SHP [25]. In addition, previous studies have shown that the JNK/MAPK pathway regulates the TGF-ß1 induced and Smad2-dependent IL-6 production in human bronchial epithelial cells [26]. Many studies have suggested that the JNK pathway contributes to the fibrotic process in different models of disease [27], [28]. These results indicate that the JNK pathway may play an important role in TGF-ß-mediated fibrosis. We also observed that ERK1/2, JNK activation was induced within several hours of cisplatin administration to HK-2 cells, and was attenuated by FXR ligand. In addition, cisplatin-induced overexpression of JNK was not attenuated by FXR ligand in SHP-knockdown HK-2 cells. These data suggest FXR ligand regulates the JNK-TGF-ß1 pathway via SHP induction. TGF-ß1 signals are transduced by transmembrane serine/threonine kinase type I and type II receptors and intracellular mediators known as Smads. Upon TGF-ß1 stimulation, receptor-bound Smad proteins such as Smad-2/3 are phosphorylated. Phosphorylation induces the association of Smad-2/3 with Smad-4, a member of the co-Smad subfamily, and they form transcriptionally active complexes that translocate into the nucleus and activate the transcription of TGF-ß-induced target genes. Smad signaling can also be negatively controlled by the inhibitory Smad-6 and Smad-7 proteins. Therefore, TGF-ß1-induced fibrosis is thought to be mediated by Smad signaling [29]. In our study, cisplatin induced overexpression of Smad-4, whereas inhibitory Smad-6 expression decreased in response to cisplatin. These cisplatin-induced changes were abolished or significantly attenuated by co-treatment with FXR ligand. In addition, FXR ligand co-treatment counteracted cisplatin-induced upregulation of fibronectin and connective tissue growth factor (CTGF) as well as epithelial mesenchymal transition. We suggest TGF ß1/Smad signaling-mediated cisplatin-induced renal fibrosis is effectively prevented by FXR ligand.

In cisplatin-induced nephrotoxicity, tubular cell apoptosis precedes the manifestations of tubular atrophy, tubular dilatation, and perivascular inflammation. Cisplatin induces Bax aggregation and translocation to the mitochondria, causing activation of caspase-9, which then cleaves and activates the effector caspase, caspase-3, leading to a loss of mitochondrial transmembrane potential and to apoptotic cell death [2]. In this study, the number of TUNEL-positive cells increased after cisplatin treatment. Along with these changes, cisplatin increased expression of Bax and downregulated expression of the anti-apoptotic protein Bcl-2. These changes were reversed by FXR ligand. Several reports have implicated MAPK pathways in apoptotic signaling by TGF-ß. For example, activation of TGF-ß–activated kinase-1 (TAK-1), a protein of the MAP kinase family, activates p38 and JNK signaling in TGF-ß family–induced apoptosis [30]. Inhibition of TGF-ß1/MAPK signaling and apoptosis by 6ECDCA suggests FXR ligand prevents apoptosis and tubular atrophy through inhibition of the TGF-ß/MAPK pathways.

### Conclusions

Our study shows that FXR ligand 6ECDCA prevented cisplatin-induced kidney injury by inhibiting renal inflammation, fibrosis, and apoptosis through the induction of SHP. This knowledge may lead to an important new therapeutic target for the treatment of kidney disease associated with cisplatin- induced nephrotoxicity.
